# Supplementary material for: Can Homecare Chronic Respiratory Disease Patients with Home Oxygen Treatment (HOT) in Southern Okinawa, Japan Be Evacuated Ahead of the Next Anticipated Tsunami?
Source: Int J Environ Res Public Health. 2022 May 6;19(9):5647. doi: 10.3390/ijerph19095647 (PMC9103670; doi:10.3390/ijerph19095647)
Supplement: Supplementary file 1 [file ijerph-19-05647-s001.zip › ijerph-1694114-supplementary.pdf]

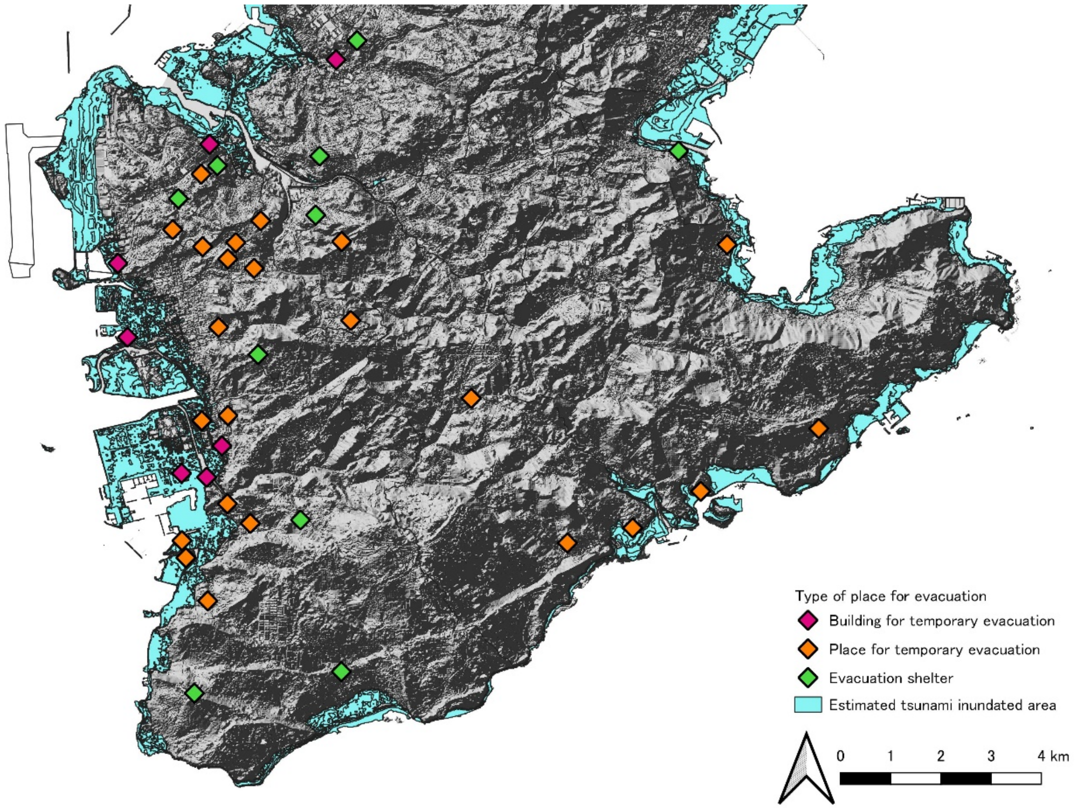

Figure S1. Locations and types of shelters in the southern areas of the main island of Okinawa examined in this study. Type of place for evacuation. Red square: Building for temporary evacuation. Orange square: Place for temporary evacuation. Green square: Evacuation shelter. Light blue area: Estimated Tsunami inundated area.
